# Role of ursodeoxycholic acid in neonatal indirect hyperbilirubinemia: a systematic review and meta-analysis of randomized controlled trials

**DOI:** 10.1186/s13052-022-01372-w

**Published:** 2022-10-17

**Authors:** Glen Lazarus, Jerrell Francie, Rosalina Dewi Roeslani, Siti Rizny Fitriana Saldi, Hanifah Oswari

**Affiliations:** 1 Gastrohepatology Division, Department of Child Health, Faculty of Medicine, Dr. Cipto Mangunkusumo Hospital, Universitas Indonesia, Jakarta, Indonesia; 2 Neonatology Division, Department of Child Health, Faculty of Medicine, Dr. Cipto Mangunkusumo Hospital, Universitas Indonesia, Jakarta, Indonesia; 3 Clinical Epidemiology and Evidence-Based Unit (CEEBM), Dr. Cipto Mangunkusumo Hospital - Faculty of Medicine Universitas Indonesia, Jakarta, Indonesia

**Keywords:** Neonatal jaundice, Neonatal hyperbilirubinemia, Ursodiol, URSODEOXYCHOLIC ACID, Phototherapy

## Abstract

**Background:**

Neonatal jaundice is a transitional phenomenon affecting three out of five full-term newborns globally. Ursodeoxycholic acid could be beneficial in neonatal jaundice needing phototherapy.

**Methods:**

We searched PubMed, EBSCO, ProQuest, and Cochrane Library up to August 21^st^, 2021, for articles to be reviewed. Meta-analysis using random-effects model was performed.

**Results:**

Eight studies involving 1116 neonates were chosen in this review; however, only five studies were included for meta-analysis. Phototherapy duration was significantly lower in the interventional group with high heterogeneities. Subgroup analysis of the phototherapy duration based on the risk of bias resulted in a shorter duration (mean difference (MD) = –17.82; 95% CI = –20.17 to –15.47; *p* =  < 0.001) with low heterogeneity in the treatment group. Secondary outcome focusing on mean total serum bilirubin showed a lower mean total serum bilirubin in 48 h post-treatment (MD = –0.43; 95% CI = –0.64 to –0.22; *p* =  < 0.0001) with low heterogeneities in Asian countries.”

**Conclusions:**

Ursodeoxycholic acid might be considered as a novel adjuvant therapy in neonatal indirect hyperbilirubinemia to shorten the phototherapy duration and lower the mean total serum bilirubin.

**Supplementary Information:**

The online version contains supplementary material available at 10.1186/s13052-022-01372-w.

## Introduction

Neonatal jaundice is a condition of hyperbilirubinemia that manifests as a yellowish discoloration of the skin, sclera, and mucous membranes in the first week of life. This transitional phenomenon affects at least three out of five full-term newborns [1, 2]. Hyperbilirubinemia itself can cause neurotoxicity by influencing central nervous system development [3, 4]. Treatment is primarily focused on using phototherapy or exchange transfusion as indicated to the individual’s bilirubin level according to their postnatal age [5–7]. Some side effects from phototherapy include interrupted maternal-neonatal interaction, water loss, electrolyte imbalance, and bronze baby syndrome; while exchange transfusion may cause thrombocytopenia, hypocalcemia, and hypokalemia [8, 9].

Novel adjuvant treatments in neonatal indirect hyperbilirubinemia are needed to increase bilirubin clearance, decrease phototherapy duration, and decrease exchange transfusion rate. Studies have shown that baby massage, intravenous fluid supplementation, several agents like fenofibrate and zinc sulfate could help ameliorate neonatal hyperbilirubinemia [10–13], although some of these options are not helpful in the acute phase because they need four days to show benefit on bilirubin concentration [10] or had no significant impact on phototherapy duration [11, 13]. Ursodeoxycholic acid (UDCA), or ursodiol, is a bile acid commonly used to manage cholestatic liver disease [14–16]. UDCA helps in improving endogenous bile secretion, displacement of more toxic components of endogenous bile acids, and reducing enterohepatic circulation. UDCA also exerts neuroprotective and hepatoprotective properties through its anti-apoptotic, anti-inflammatory, and antioxidant effects [17–20]. UDCA has also been investigated for its possible role in indirect hyperbilirubinemia. One randomized clinical trial by Honar et al*.* revealed that UDCA could reduce the duration of phototherapy, hospitalization period, and the mean bilirubin in the intervention group was significantly lower than the control group [21]. Another trial by Mirzarahimi et al. showed that the addition of UDCA provided no significant difference compared to phototherapy alone [22].

We performed a systematic review of randomized clinical trials to investigate the possible role of ursodeoxycholic acid in treating neonatal indirect hyperbilirubinemia because previous studies had shown conflicting results [21, 22]. Meta-analysis will also be done to pool and evaluate the existing evidence.

## Methods

We followed the checklist recommended by the Preferred Reporting Items for Systematic Reviews and Meta-Analyses flow diagram (PRISMA) guidelines [23]. The protocol was registered on PROSPERO (registration number: CRD42021266663).

### Types of studies

We included randomized clinical trial studies regarding the role of ursodeoxycholic acid in the cases of neonatal jaundice. Observational studies, case series and full-text articles using a language other than English were excluded from this systematic review.

### Types of participants

Term neonates (born ≥ 37 weeks) younger than 14 days old were included. Studies with preterm neonates, sepsis, glucose-6-phosphate dehydrogenase deficiency, rhesus incompatibility, infants of diabetic mothers, cholestasis, and congenital anomalies were excluded.

### Type of intervention and control

Studies using UDCA at any dose and duration in addition to routine phototherapy for pathological neonatal jaundice that was compared to control (routine phototherapy only) were included. Phototherapy was done when the total serum bilirubin exceeded the line indicated for the neonate’s age [7].

### Types of outcomes

The primary outcome is the phototherapy duration needed to reach the desirable bilirubin level (in hours). The secondary outcomes include exchange transfusion rate (as percentage or number of patients); side effects (including diarrhea or vomiting; reported as percentage or number of patients); and the serum bilirubin level per 12 h (as mean bilirubin level and/or mean changes of bilirubin level compared to the baseline, reported in mg/dL).

We searched the following databases: PubMed, EBSCO, ClinicalTrials.gov, and Cochrane Library (from inception to August 21^st^, 2021). The keywords used included variations for the spelling of ursodeoxycholic acid and neonatal jaundice. Further searches were done from the reference lists of included studies. Search terms for PubMed are provided in Supplementary Table 1.

### Selection of studies

Two researchers (GL and JF) independently screened the titles and abstracts from each database. Selected articles were independently read for full-text review and reviewed for eligibility. Disagreements were solved through consultation with a third member of the review team (HO).

### Data extraction

The following information were extracted: author, number of patients, age at admission, female gender, weight, etc. All the data were independently extracted and entered in Microsoft Excel spreadsheets (GL, JF), compared and differences resolved by discussion, or if required, consultation with a third member of the review team (HO). We contacted the corresponding authors of the included studies by email to confirm the missing data or methodological information, had they not been reported sufficiently in the studies.

### Study risk of bias assessment

Two authors (GL and JF) evaluated the risk of bias of each included study by using the Cochrane RCT risk-of-bias tool using Review Manager (RevMan) Software version 5.4 [24]. When two authors disagreed, a third reviewer was involved until a consensus was reached. For this review, we consider randomization as a key element in assessing the overall risk of bias.

### Synthesis methods

We performed a meta-analysis with Review Manager (RevMan) Software version 5.4 from Cochrane Collaboration using the random-effects model. Heterogeneity between studies was assessed using the *χ*^2^ test and the amount of variation was estimated by calculating the *I*^2^. Heterogeneity with *p* < 0.05 and *I*^2^ > 50% were considered heterogeneous. Subgroup analyses were then performed if there was a need to investigate the possible source of heterogeneity.

### Certainty assessment

The quality of the body of evidence that contributes data to the meta-analyses was assessed using the GRADE methodology (GRADEpro, Version 20. McMaster University, 2014).The certainty level for each body of evidence will be presented in Supplementary Table 3.

## Results

### Studies included in systematic review and meta-analysis

**Fig. 1:**
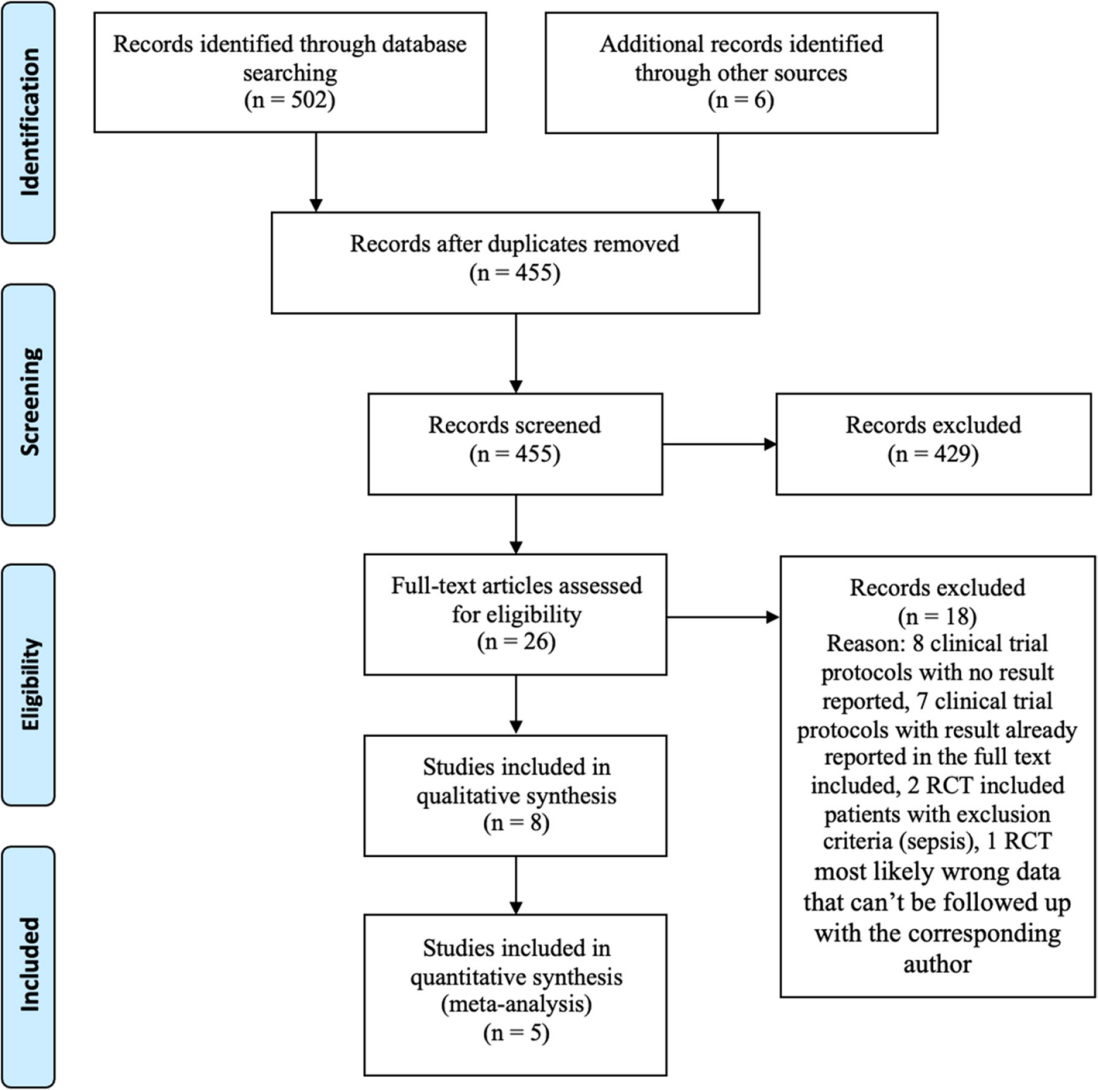
PRISMA flow diagram. The PRISMA (Preferred Reporting Items for Systematic Reviews and Meta-Analysis) flow diagram demonstrates our study selection process

A detailed PRISMA flow diagram for the study selection process is shown in Fig. 1. We initially retrieved 508 studies from four databases and other sources, 53 articles were excluded based on duplicated articles, 429 articles were excluded based on title and abstract that did not contain data on UDCA and neonatal hyperbilirubinemia, review articles, and conference abstracts with no abstract mentioning UDCA and neonatal jaundice. Another 18 articles were excluded after reading the full text, eight of which were clinical trial protocols with no result reported, seven articles were clinical trial protocols with results already reported in the included studies, two studies included patients with our exclusion criteria (sepsis), and one study with irregular data that couldn’t be followed up with the corresponding author. Consequently, eight studies were included in the systematic review [21, 25–31]. The characteristics of the included studies are displayed in Tables 1, 2 and 3.

**Table 1:** Characteristics of included studies (Part 1)

		UDCA supplementation group (UDCA + phototherapy)						Control group (phototherapy only)				
No	Author	Number of participants	Mean age (SD)	Female (n)	Weight (g)	TSB (mg/dL)	Therapy	Number of participants	Mean age (SD)	Female (n)	Weight (g)	TSB (mg/dL)
1	Akefi [25]	110	5.3 (2.9)	62	–	16.8 (2.4)	UDCA 10 mg/kg/d	110	4.9 (2.1)	52	–	15.7 (2.5)
2	El-Gendy [26]	50	4,9 (1.44)	20	–	16.5 (1.51)	UDCA 1*	50	4.86 (1.6)	24	–	16.4 (1.57)
3	Gharehbaghi [27]	80 (A = 40, B = 40)	A = 5.91 (2.49), B = 5.1 (2.47)	A = 18, B = 16	A = 3175.41 (561.53), B = 2961.71 (440.03)	A = 19.88 (2.33), B = 19.33 (2.51)	A = UDCA 1* B = UDCA 2*	40	5.91 (2.49)	18	3192.91 (431.02)	19.76 (2.64)
4	Hassan [28]	100	5.4 (1.4)	44	3.2 (0.4) (in kg)	16.3 (1.7)	UDCA 1*	100	5.3 (1.5)	49	3.1 (0.4) (in kg)	16.5 (2.9)
5	Honar [21]	40	3.7 (1)	21	2970 (292)	15.9 (1.7)	UDCA 1*	40	3.6 (1)	22	2985 (312)	16.3 (1.5)
6	Jafari [29]	64 (A = 32, B = 32)	–	–	–	A = 16.47 (1.65), B = 16.22 (1.67)	A = UDCA 1* B = UDCA 3*	32	–	–	–	16.64 (1.72)
7	Meena [30]	50	–	19	2.73 (0.23)	16.33 (1.58)	UDCA 1*	50	–	17	2.69 (0.41)	15.6 (2.78)
8	Shahramian [31]	100	–	47	–	–	UDCA 2*	100	–	48	–	–

UDCA 1* UDCA 10 mg/kg/day divided every 12 h, UDCA 2* UDCA 15 mg/kg/day divided every 12 h, UDCA 3* UDCA 20 mg/kg/day divided every 12 h

**Table 2:** Characteristics of included studies (Part 2). Outcomes reported

No	Author	PT duration	TSB 12 h (mg/dL)	TSB 24 h (mg/dL)	TSB 36 h (mg/dL)	TSB 48 h (mg/dL)	Side effects	Exchange transfusion	Other outcomes reported
1	Akefi [25]	+	+	+	–	–	+	–	Total bilirubin level reduction (compared to baseline)
2	El-Gendy [26]	+	–	+	–	+	+	–	Mean total bilirubin at 72- and 96-h of treatment, duration of phototherapy, duration of admission
3	Gharehbaghi [27]	–	–	–	–	–	+	–	Indirect bilirubin at 6-, 12-, 24-, 48-h, and discharge, mean length of hospital stay
4	Hassan [28]	+	+	+	+	–	+	–	Total of patients still needing phototherapy at 36- and 48-h after treatment
5	Honar [21]	+	+	+	–	+	+	–	–
6	Jafari [29]	–	–	–	–	–	+	–	Mean total serum bilirubin (TSB) and mean TSB difference at 8-, 16-h post treatment, and final visit, total of patients with TSB returned to normal at 8-, 16-, 24-, and 36-h post treatment
7	Meena [30]	–	+	+	+	+	+	–	Mean difference between the two groups at 12-, 24-, 36-, and 48-h post treatment
8	Shahramian [31]	–	–	–	–	–	–	–	Ratio of neonates reaching TSB < 10 mg/dL, changes of TSB, changes of direct bilirubin levels at 24-, 48-, and 72-h from birth

*TSB* Total serum bilirubin, *PT* Phototherapy

**Table 3:** Characteristics of included studies (Part 3). Additional data

		UDCA supplementation group (UDCA + phototherapy)		Control group (phototherapy only)			
**No**	**Author**	Phototherapy duration (hours)	Last mean bilirubin reported (mg/dL)	Phototherapy duration (hours)	Last mean bilirubin reported (mg/dL)	Type of phototherapy lamp	Thresholds for phototherapy
1	Akefi [25]	34.6 (16.3)	–	35.7 (18.2)	–	Simple phototherapy	Standard curve
2	El-Gendy [26]	65.2 (12.8)	9.42 (0.82) (96 h)	82.5 (19.4)	10.5 (1.35) (96 h)	Single PT	–
3	Gharehbaghi [27]	29.47 (16.8)	4.75 (1.07) (discharge)*	45.97 (18.01)	7.88 (2.11) (discharge)*	LED	AAP guidelines, termination: decrease in TSB < 50% exchange threshold or TSB < 10 mg/dl
4	Hassan [28]	23.2 (5.6)	7.6 (0.9) (36 h)	41.1 (7.2)	9.1 (0.8) (48 h)	LED	–
5	Honar [21]	15.5 (6)	9.8 (0.2) (48 h)	44.6 (13.3)	10.1 (1.1) (48 h)	Daylight fluorescent	Until TSB < 10 mg/dL
6	Jafari [29]	–	14.61 (2.29) (16 h)	–	12.58 (2.25) (36 h)	–	Until TSB normalised
7	Meena [30]	–	9.48 (0.68) (48 h)	–	9.99 (0.66) (48 h)	LED	Until TSB < 10 mg/dL
8	Shahramian [31]	–	–	–	–	Daylight fluorescent	Until TSB < 10 mg/dL

*TSB* Total serum bilirubin, *PT* Phototherapy

*Reported as indirect bilirubin in mg/dL

Three studies included neonates younger than 14 days old as the inclusion criteria [28, 29, 31], two studies reported their age range was 3–7 days old [21, 26], two studies reported their mean age was 4.9–5.91 days [25, 27], and one study did not report patient’s mean age [30]. All eight articles were randomized clinical trials done in various countries (four studies in Iran, two studies in India, one study in Egypt, and one study in Iraq). They were all published between 2015 and July 2021, comparing outcomes between intervention groups using UDCA in addition to phototherapy and control groups using phototherapy only.

Due to different reported outcomes, three studies were not included in the meta-analysis. The outcomes were: indirect bilirubin (not total serum bilirubin) [27], incomplete total serum bilirubin reported (not measured after reaching normal value) [29], and changes of direct bilirubin [31]. The summary of these three studies’ findings is presented in Table 4.

**Table 4:** Results summary of studies not included in meta-analysis

Author	Summary
Gharehbaghi [27]	- UCB levels at 6-, 12-, 24-, 48-h, and discharge were lower in patients receiving UDCA than control group (*p* < 0.05) - UCB levels at 6-, 12-, 24-, and 48-h were lower in patients receiving higher doses of UDCA (15 mg/kg/day vs 10 mg/kg/day) (*p* < 0.05) - Mean length of hospital stay was 29.47 ± 16.8 h (group receiving UDCA 10 mg/kg/day), 21.35 ± 8.12 h (group receiving UDCA 15 mg/kg/day), and 45.97 ± 18.01 h (control group) (*p* < 0.05)
Jafari [29]	- Mean TSB after 8 h was significantly different between three groups (control vs group receiving UDCA 10 mg/kg/day vs group receiving UDCA 20 mg/kg/day) (*p* < 0.05) - Mean TSB difference was significant after 8 h of therapy in control group versus intervention group - Phototherapy could be stopped at 16 h post treatment in 56% (control group), 97% (group receiving UDCA 10 mg/kg/day), and 100% (group receiving UDCA 20 mg/kg/day)
Shahramian [31]	- The ratio of neonates reaching TSB < 10 mg/dL was higher in the phototherapy + UDCA group at 48-, and 72-h after birth (*p* < 0.05) - Mean TSB differences at 48- and 72-h after birth were higher in phototherapy + UDCA group (*p* < 0.05) - Changes of direct bilirubin were no significant during 48- and 72-h in both groups

**Fig. 2:**
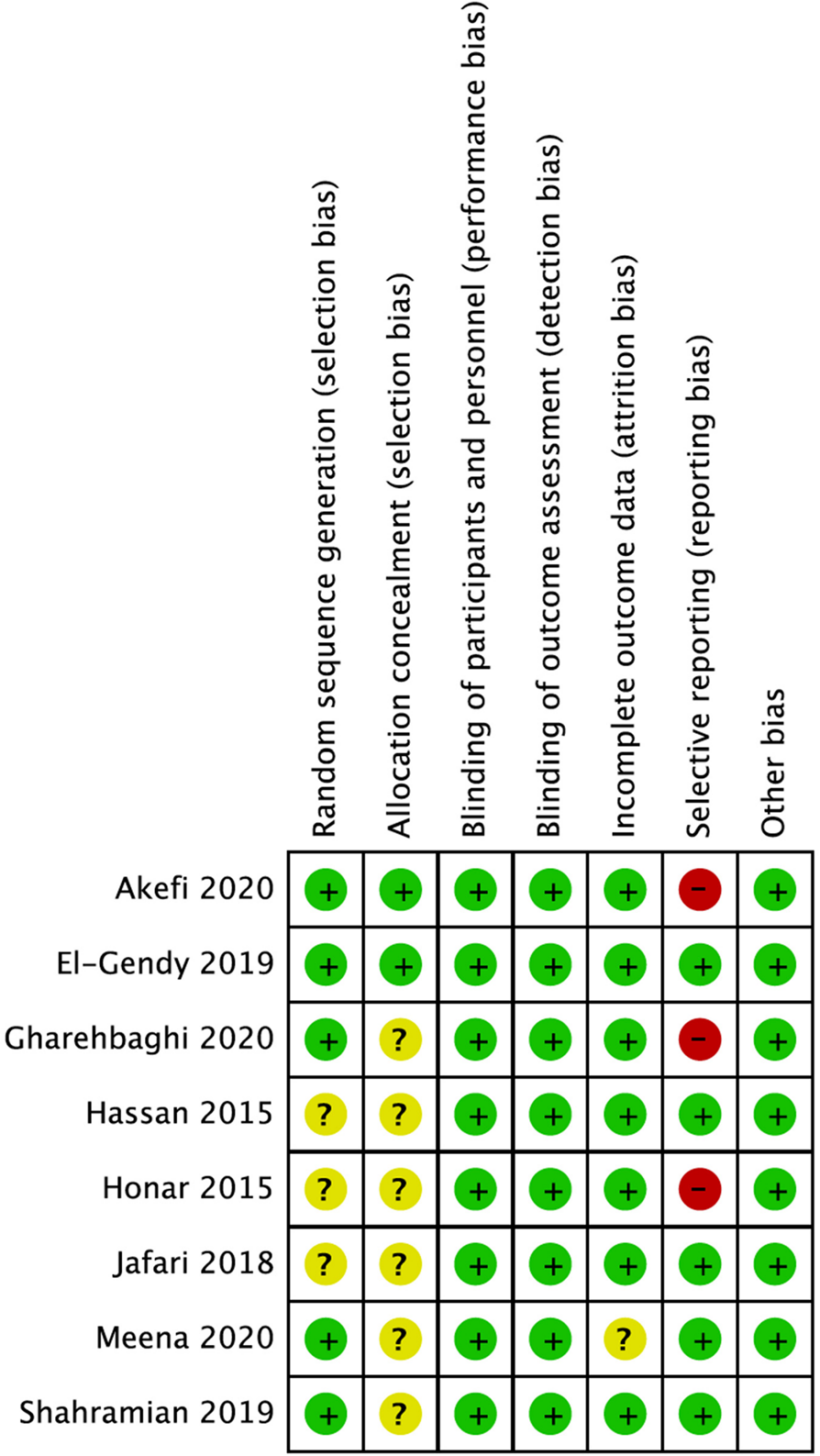
Risk of bias summary. Review authors' judgements about each risk of bias item for each included study

The risk of bias was low to moderate for all studies (See Fig. 2). Randomization was done in all studies, but three studies did not specify their randomization method [21, 28, 29]. Six out of eight studies showed an unclear selection bias because of not having detailed allocation concealment. Reporting bias was also detected in four studies [21, 25, 27]. Attempts to contact the corresponding authors via email on the studies mentioned above and those with incomplete data were made, but there had been no reply. A supporting explanation for the risk of bias assessment is provided in Supplementary Table 2.

### Primary outcomes

**Fig. 3:**
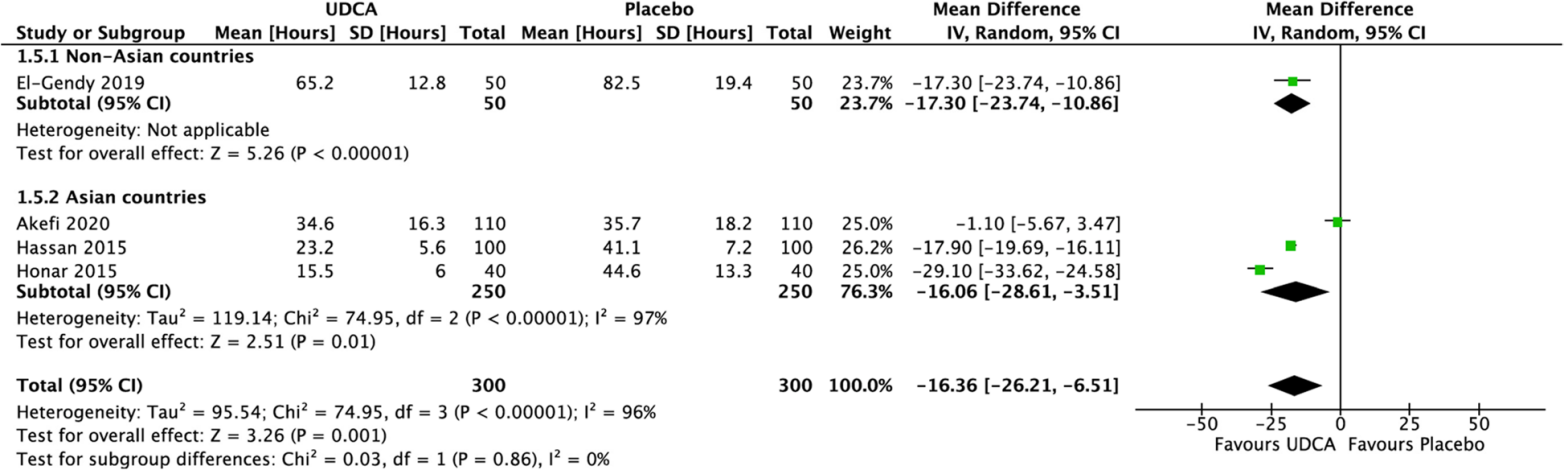
Forest plot for the meta-analysis of duration of phototherapy

**Fig. 4:**
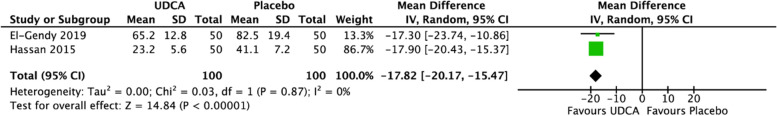
Forest plot for the meta-analysis of duration of phototherapy based on risk of bias

Figure 3 showed that adding UDCA could result in a lower duration of phototherapy than control groups (mean difference (MD) = –16.36 h; 95% CI = –26.21 to –6.51; *p* = 0.001) with significant heterogeneity (*I* ^2^ = 96%, *p* =  < 0.00001) and low certainty evidence (downgraded due to inconsistency and imprecision). Subgroup analysis for phototherapy duration based on Asian countries also showed a high heterogeneity (*I*^2^ = 97%, *p* < 0.00001), while subgroup analysis based on the risk of bias showed that adding UDCA in El-Gendy et al. [26] and Hassan et al. [28] studies had a significantly lower duration of phototherapy (mean difference (MD) = –17.82 h; 95% CI = –20.17 to –15.47; *p* =  < 0.001) with a low heterogeneity (*I* ^2^ = 0%, *p* = 0.86) and a high certainty level (See Fig. 4).

### Secondary outcomes

**Fig. 5:**
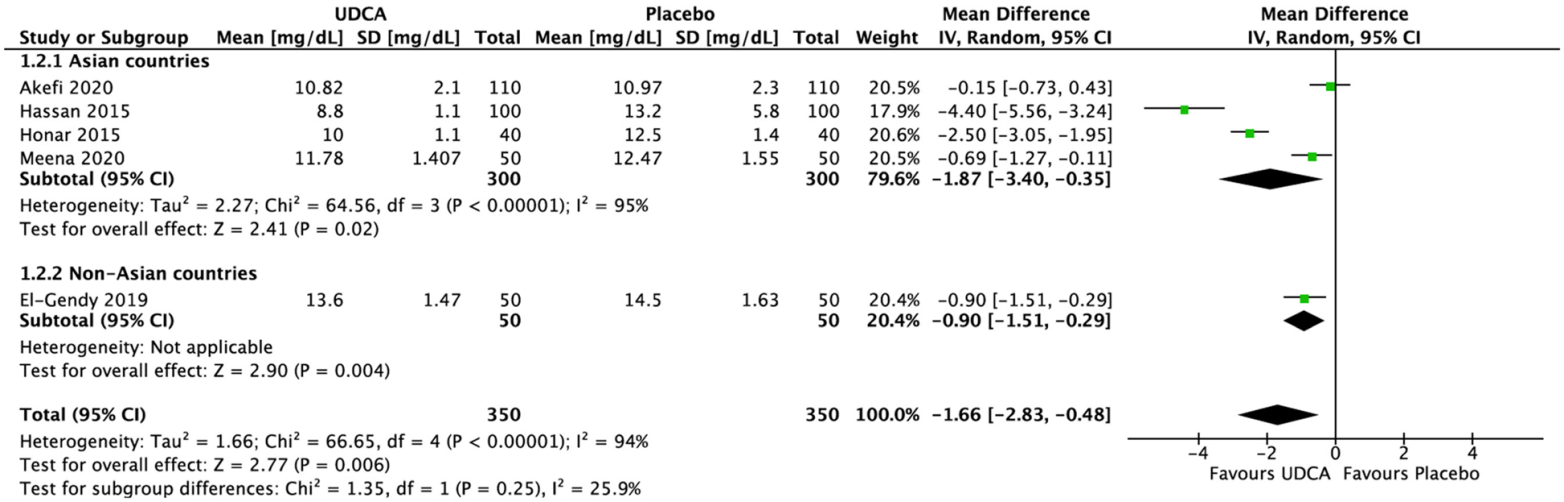
Forest plot for the meta-analysis of TSB at 36 h

**Fig. 6:**
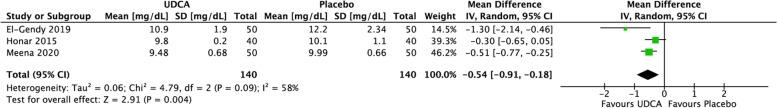
Forest plot for the meta-analysis of TSB at 48 h

**Fig. 7:**
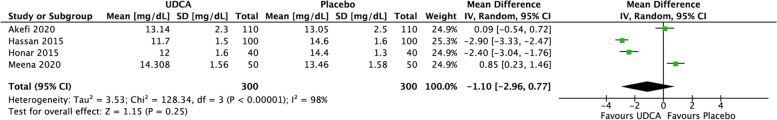
Forest plot for the meta-analysis of TSB at 12 h

**Fig. 8:**
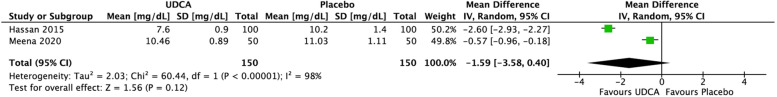
Subgroup analysis of mean TSB at 48 h between UDCA and control group. Subgroup analysis was done for Asian vs non-Asian countries

**Fig. 9:**
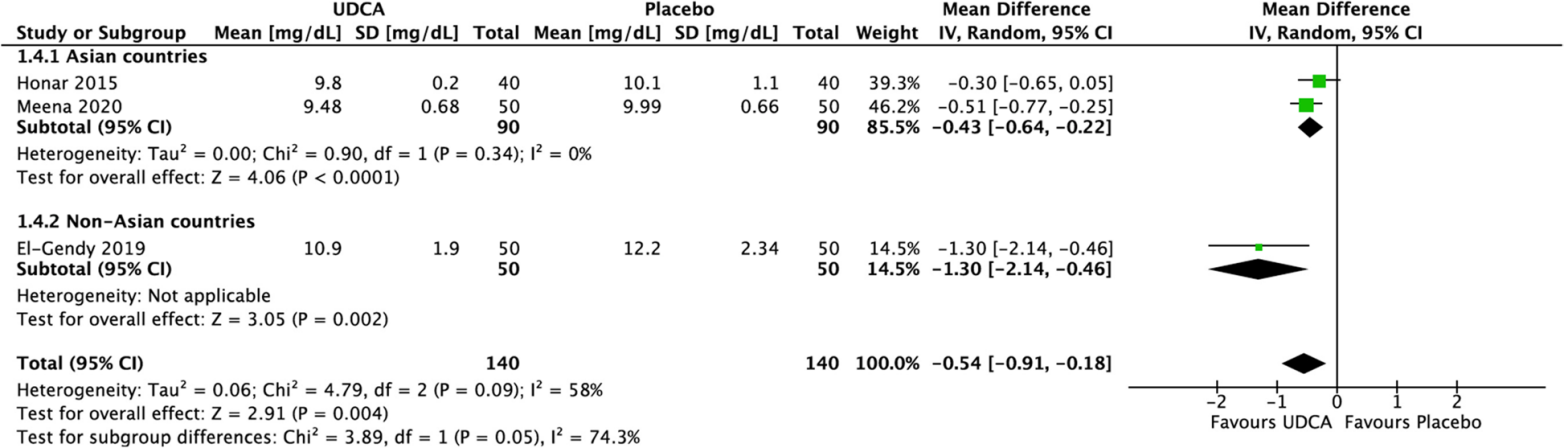
Forest plot for the meta-analysis of TSB at 24 hours

TSB comparison between intervention and control groups using the random-effect model was presented in Figs. 5, 6, 7, 8 and 9. Statistically significant results were found from meta-analyses comparing TSB in UDCA and control groups at 24 h (MD = –1.66 mg/dL; 95% CI = –2.83 to –0.48; *p* = 0.006) with high heterogeneity (*I* ^2^ = 94%, *p* < 0.00001, Fig. 5) and 48 h (MD = –0.54 mg/dL; 95% CI = –0.91 to –0.18; *p* = 0.004) with moderate heterogeneity (*I*^2^ = 58%, *p* = 0.09, Fig. 6), while meta-analyses at 12 h (MD = –1.10 mg/dL; 95% CI = –2.96 to 0.77; *p* = 0.25, Fig. 7) and 36 h (MD = –1.59 mg/dL; 95% CI = –3.58 to 0.40; *p* = 0.12, Fig. 8) showed nonsignificant mean TSB difference between the two groups. GRADE assessment for all outcomes (TSB at 12-, 24-, 36-, and 48-h post treatment) was low certainty (downgraded due to inconsistency and imprecision), while subgroup analysis at the 48 h for Asian countries resulted in a moderate certainty evidence (downgraded due to imprecision). Subgroup analysis for TSB at 12- and 36-h post treatment was not done because all the studies were from Asian countries, while subgroup analysis for TSB at 24-h post treatment also showed high heterogeneity (*I*^2^ = 95%, *p* < 0.00001).

Two studies [25, 31] also described the mean changes of serum bilirubin between interventional and control groups. Significantly faster bilirubin clearance in the group using UDCA could be seen in a study by Akefi et al. [25] on the 12 h after phototherapy started (3.7 mg/dL vs 2.7 mg/dL, *p* = 0.001) and Shahramian et al. [31] on the 48 and 72 h post-treatment. While data on 24 h after phototherapy started showed higher mean TSB difference in the UDCA group, although this finding was not statistically significant in Shahramian et al. study.

Subgroup analyses were performed to investigate study heterogeneity. Subgroup analyses based on sex were not conducted because the data were unavailable. Based on the data available of the included studies, subgroup analyses were conducted to separate studies done in Asian countries and non-Asian countries. Stratifying by Asian and non-Asian countries, neonates receiving UDCA in addition to phototherapy had lower mean TSB at 48 h than the control group in Asian countries [21, 30] (mean difference (MD) = –0.43 mg/dL; 95% CI = –0.64 to –0.22; *p* =  < 0.0001) with low heterogeneities (*p* = 0.34, *I*^2^ = 0%) (See Fig. 9).

The observation from six studies showed that there are no side effects of UDCA administration (e.g. diarrhea and vomiting) [21, 25, 26, 28–30] and one study stated that the diarrhea in the UDCA group was seen more often but not significantly more than in the control group [27]. No study examined the effect of adding UDCA to phototherapy on the exchange transfusion rate.

## Discussion

Previous studies had shown conflicting results on phototherapy duration and TSB, which could be caused by other factors influencing the phototherapy’s effectiveness that unfortunately were not described completely in the included studies. A recent meta-analysis by Kuitunen et al. showed that adding UDCA to usual therapy resulted in bigger TSB reduction and faster phototherapy duration but the result showed high heterogeneities among the studies, thus making it difficult to interpret. Our review showed that adding UDCA as an adjuvant to the phototherapy of neonatal hyperbilirubinemia resulted in a lower phototherapy duration and faster TSB decline.

Our meta-analysis showed that adding UDCA could result in 17.82 (15.47 – 20.17) hours of faster phototherapy duration than the control group. Two studies [21, 28] showed up to halved reductions of phototherapy duration compared to other studies, which could be caused by different exclusion criteria and different light intensity or spectral emission that was not described. Subgroup analysis based on the risk of bias was done in order to lower the heterogeneity between the studies on phototherapy duration outcome. Lowering the phototherapy duration might lower the phototherapy exposure, side effects, hospitalization, and cost [32]. Phototherapy may have some side effects, but some of these are more common in conventional phototherapy devices, for example by using fluorescent tubes [33]. Therefore, providing the information of phototherapy device used in the randomized trials is important to make sure future adjunctive therapy recommendations are properly made. Unfortunately, some studies didn’t provide the lamp details [25, 26, 30] so it was not possible to do a subgroup analysis based on what phototherapy lamp used.

Our secondary outcome found that the addition of UDCA to the standard phototherapy resulted in a lower mean TSB in 24- and 48-h post-treatment with high heterogeneities. Subgroup analyses revealed that Asian and non-Asian countries (particularly African) influenced the effect of UDCA in neonatal indirect hyperbilirubinemia in the 48-h post-treatment (mean difference (MD) = –0.33; 95% CI = –0.60 to –0.07; *p* = 0.01) with lower heterogeneities (*p* = 0.19, *I*^2^ = 39%). This difference may be attributed to the different bilirubin metabolism genes and different melanosome characteristics that could contribute to higher TSB. Melanosomes in dark skin are larger and more heavily pigmented, which could reduce the penetration of light [34–36]. It should also be highlighted that some studies [21, 30, 31] only test their serum bilirubin until below 10 mg/dL, causing the serum bilirubin reported was around this cut-off and previously already normal neonates were not included in the next blood tests anymore. Insignificant statistic power of UDCA intervention in neonatal indirect hyperbilirubinemia on the 36 h post-treatment (*p* = 0.20) could be caused by the different amount of study included. There were only two studies that examined total serum bilirubin levels in the 36 h post-treatment [28, 30]. It is interesting to note that all of the RCTs included had been performed in lower- or middle-income countries (LMICs), which is in accordance with the fact that they have higher neonatal jaundice’s prevalence and mortality that could be caused by limited access to diagnostic evaluation, conventional treatment, and inadequate phototherapy devices’ function [36, 37].

**Fig. 10:**
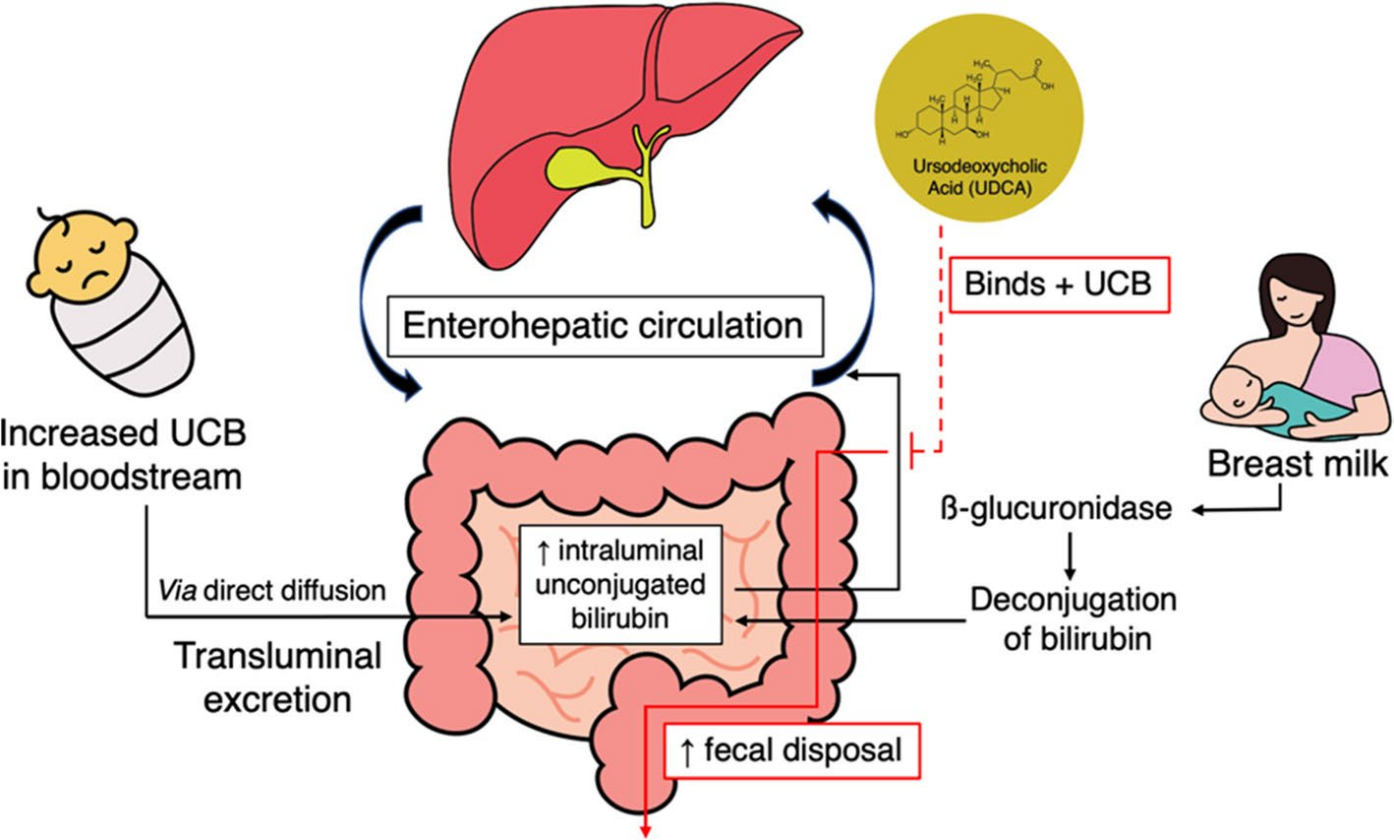
Role of ursodeoxycholic acid (UDCA) in indirect hyperbilirubinemia. UCB = unconjugated bilirubin; red lines = UDCA mechanism of action

The role of UDCA in reducing UCB could be explained by a study by Cuperus et al. [38]. The study showed that UDCA administration trapped UCB that was excreted to the intestinal lumen via direct diffusion during indirect hyperbilirubinemia condition. The efficiency of UCB’s direct diffusion to the intestinal lumen was decreased by several things. First, neonates have insufficient intestinal anaerobic flora, even though these floras could help in reducing the UCB entering the enterohepatic circulation by converting it to urobilinoids. Second, neonatal liver and intestinal immaturity could result in slower removal of bilirubin. The UCB burden is also increased by breast milk β-glucuronidase activity, which deconjugates the intestinal direct bilirubin back to UCB [39–41]. The administration of UDCA could bind the UCB content in intestinal lumen, preventing it from entering the enterohepatic circulation, and increasing its disposal through faeces (see Fig. 10) [38].

The varied locations, methods, risk of bias, and reporting methods could be considered the major limitation from this review, which could result in significant heterogeneities among the meta-analysis results, but these issues were solved by doing subgroup analyses. Although our primary outcome showed a high certainty level, there are still many other outcomes with low certainty. Future studies should focus and provide complete data on: phototherapy details (duration, tools, intensity, etc.), monitoring side effects (both from the administration of UDCA and the phototherapy), exchange transfusion rate, and others.”

## Conclusion

Our meta-analysis suggests that the addition of ursodeoxycholic acid (UDCA) to phototherapy could reduce phototherapy duration by almost 18 h compared to phototherapy only in low risk of bias studies. It also resulted in a lower mean of total serum bilirubin in the 48 h post-treatment, especially in Asian countries. UDCA administration might be considered an adjuvant therapy in neonatal jaundice, considering that UDCA is safer, cheaper, and more applicable in clinical settings.

## Supplementary Information

**Additional file 1:** **Supplementary Table 1.** PubMed search terms. **Supplementary Table 2.** Risk of bias for included studies. **Supplementary Table 3.** GRADE Assessment.

## Data Availability

The datasets generated and analysed during the current study are available from the corresponding author on reasonable request.

## Abbreviations

PT: Phototherapy
RCT: Randomized controlled trial
TSB: Total serum bilirubin
UCB: Unconjugated bilirubin
UDCA: Ursodeoxycholic acid
