# Supplementary material for: Role of ursodeoxycholic acid in neonatal indirect hyperbilirubinemia: a systematic review and meta-analysis of randomized controlled trials
Source: Ital J Pediatr. 2022 Oct 17;48:179. doi: 10.1186/s13052-022-01372-w (PMC9575272; doi:10.1186/s13052-022-01372-w)
Supplement: Supplementary file 1 — Additional file 1: Supplementary Table 1. PubMed search terms. Supplementary Table 2. Risk of bias for included studies. Supplementary Table 3. GRADE Assessment. [file 13052_2022_1372_MOESM1_ESM.docx]

**Supplementary Table 1. PubMed Search Terms**

| ("ursodeoxycholic acid"[MeSH Terms] OR ("ursodeoxycholic"[All Fields] AND "acid"[All Fields]) OR "ursodeoxycholic acid"[All Fields] OR ("ursodeoxycholic acid"[MeSH Terms] OR ("ursodeoxycholic"[All Fields] AND "acid"[All Fields]) OR "ursodeoxycholic acid"[All Fields] OR "ursodiol"[All Fields])) AND ("jaundice, neonatal"[MeSH Terms] OR ("jaundice"[All Fields] AND "neonatal"[All Fields]) OR "neonatal jaundice"[All Fields] OR ("neonatal"[All Fields] AND "jaundice"[All Fields]) OR ("neonatal hyperbilirubinaemia"[All Fields] OR "hyperbilirubinemia, neonatal"[MeSH Terms] OR ("hyperbilirubinemia"[All Fields] AND "neonatal"[All Fields]) OR "neonatal hyperbilirubinemia"[All Fields] OR ("neonatal"[All Fields] AND "hyperbilirubinemia"[All Fields])) OR ("jaundice, neonatal"[MeSH Terms] OR ("jaundice"[All Fields] AND "neonatal"[All Fields]) OR "neonatal jaundice"[All Fields] OR ("icterus"[All Fields] AND "neonatorum"[All Fields]) OR "icterus neonatorum"[All Fields]) OR (("indirect"[All Fields] OR "indirects"[All Fields]) AND ("hyperbilirubinaemia"[All Fields] OR "hyperbilirubinemia"[MeSH Terms] OR "hyperbilirubinemia"[All Fields] OR "hyperbilirubinaemias"[All Fields] OR "hyperbilirubinemias"[All Fields])) OR ("unconjugated"[All Fields] AND ("hyperbilirubinaemia"[All Fields] OR "hyperbilirubinemia"[MeSH Terms] OR "hyperbilirubinemia"[All Fields] OR "hyperbilirubinaemias"[All Fields] OR "hyperbilirubinemias"[All Fields])))  **Search Engine: PubMed**  **Search Date: August 21^st^ , 2021**  **Articles Identified: 61** |
| --- |

**Supplementary Table 2. Risk of Bias for Included Studies**

**2.1. Akefi *et al*., 2020 (25)**

| **Bias** | **Authors' judgement** | **Support for judgement** |
| --- | --- | --- |
| Random sequence generation (selection bias) | Low risk | Quote: "... neonates were randomly assigned into two equal groups by permuted balanced block randomization method with block size 4 ..." |
| Allocation concealment (selection bias) | Low risk | Quote: "By an online application called “Sealed Envelope”, ..." |
| Blinding of participants and personnel (performance bias) | Low risk | Quote: "In this study, the parents and the outcomes assessor were both blinded to allocation of patients to the groups."  Comment: Although blinding of personnel was not specified, the possible subjectivity would unlikely affect the outcome because it was measured objectively. |
| Blinding of outcome assessment (detection bias) | Low risk | Quote: "In this study, the parents and the outcomes assessor were both blinded to allocation of patients to the groups." |
| Incomplete outcome data (attrition bias) | Low risk | Comment: There was no lost to follow-up or discontinued intervention patient. |
| Selective reporting (reporting bias) | High risk | Comment: Study protocol's timepoint stated that there should be serum bilirubin 48 hours post-treatment, but it was not stated in the methods and results. |
| Other bias | Low risk | Comment: None were identified. |

**2.2. El-Gendy *et al.*, 2019 (26)**

| **Bias** | **Authors' judgement** | **Support for judgement** |
| --- | --- | --- |
| Random sequence generation (selection bias) | Low risk | Quote: "Randomization sequence was created using NCSS PASS 11 (11.0.8 portable, Kaysville, Utah, USA) statistical software with a 1:1 allocation using random block sizes of 50 patients per block." |
| Allocation concealment (selection bias) | Low risk | Quote: "The allocation sequence was concealed from the researcher enrolling and assessing participants in sequentially numbered, opaque, sealed, and stapled envelopes." |
| Blinding of participants and personnel (performance bias) | Low risk | Quote: "Both patient and personnel involved in assessment of the effect of UDCA in lowering neonatal indirect hyperbilirubinemia were blinded to allocation."  Comment: Although blinding was not specified, patients (in this review: neonates) couldn't differentiate whether they were in intervention or control group.. |
| Blinding of outcome assessment (detection bias) | Low risk | Quote: "Both patient and personnel involved in assessment of the effect of UDCA in lowering neonatal indirect hyperbilirubinemia were blinded to allocation." |
| Incomplete outcome data (attrition bias) | Low risk | Comment: No patient was eliminated. |
| Selective reporting (reporting bias) | Low risk | Comment: None were identified. |
| Other bias | Low risk | Comment: None were identified. |

**2.3. Gharehbaghi *et al*., 2020 (27)**

| **Bias** | **Authors' judgement** | **Support for judgement** |
| --- | --- | --- |
| Random sequence generation (selection bias) | Low risk | Quote: "The patients were assigned to the groups randomly using a random number table." |
| Allocation concealment (selection bias) | Unclear risk | Comment: Allocation concealment was not specified. |
| Blinding of participants and personnel (performance bias) | Low risk | Quote: "Throughout the study, the responsible physicians were kept blind to the grouping of the patients." and "Group C was the control group and the patients received phototherapy and the placebo." |
| Blinding of outcome assessment (detection bias) | Low risk | Quote: "Throughout the study, the responsible physicians were kept blind to the grouping of the patients." |
| Incomplete outcome data (attrition bias) | Low risk | Comment: No patient was eliminated. |
| Selective reporting (reporting bias) | High risk | Comment: Study protocol and study method's primary outcome stated that there should be total serum bilirubin measured, but the results reported were indirect bilirubin. |
| Other bias | Low risk | Comment: None were identified. |

**2.4. Hassan *et al*., 2015 (28)**

| **Bias** | **Authors' judgement** | **Support for judgement** |
| --- | --- | --- |
| Random sequence generation (selection bias) | Unclear risk | Comment: Randomization method was not mentioned. |
| Allocation concealment (selection bias) | Unclear risk | Comment: Allocation concealment was not specified. |
| Blinding of participants and personnel (performance bias) | Low risk | Comment: Although blinding of participants and personnel was not specified, the possible subjectivity would unlikely affect the outcome because it was measured objectively. |
| Blinding of outcome assessment (detection bias) | Low risk | Comment: Although blinding was not mentioned, objective outcomes (serum bilirubin and duration of phototherapy) would prevent detection bias from happening. |
| Incomplete outcome data (attrition bias) | Low risk | Comment: No patient was eliminated. |
| Selective reporting (reporting bias) | Low risk | Comment: Study protocol was not available to identify any other unreported outcomes, but the variables mentioned in methods and results were the same. |
| Other bias | Low risk | Comment: None were identified. |

**2.5. Honar *et al.*, 2015 (21)**

| **Bias** | **Authors' judgement** | **Support for judgement** |
| --- | --- | --- |
| Random sequence generation (selection bias) | Unclear risk | Comment: Randomization method was not mentioned. |
| Allocation concealment (selection bias) | Unclear risk | Comment: Allocation concealment was not specified. |
| Blinding of participants and personnel (performance bias) | Low risk | Quote: "Only 1 physician knew the code of neonates who received the UDCA and did not have any contact with either the parents or the medical team (including the nurses). Neither the nurse nor the parents knew which neonate received the UDCA." |
| Blinding of outcome assessment (detection bias) | Low risk | Quote: "Only 1 physician knew the code of neonates who received the UDCA and did not have any contact with either the parents or the medical team (including the nurses). Neither the nurse nor the parents knew which neonate received the UDCA." |
| Incomplete outcome data (attrition bias) | Low risk | Comment: No patient was eliminated. |
| Selective reporting (reporting bias) | High risk | Comment: Study protocol and study method's stated that bilirubin measurement was done every 12 hours, but the results reported were bilirubin on 12, 24, and 48 hours post treatment (there was no bilirubin reported for 36 hours). |
| Other bias | Low risk | Comment: None were identified. |

**2.6. Jafari *et al*., 2018 (29)**

| **Bias** | **Authors' judgement** | **Support for judgement** |
| --- | --- | --- |
| Random sequence generation (selection bias) | Unclear risk | Comment: Randomization method was not mentioned. |
| Allocation concealment (selection bias) | Unclear risk | Comment: Allocation concealment was not specified. |
| Blinding of participants and personnel (performance bias) | Low risk | Comment: Although blinding of participants and personnel was not specified, the possible subjectivity would unlikely affect the outcome because it was measured objectively. |
| Blinding of outcome assessment (detection bias) | Low risk | Comment: Although blinding was not mentioned, objective outcomes (serum bilirubin and duration of phototherapy) would prevent detection bias from happening. |
| Incomplete outcome data (attrition bias) | Low risk | Comment: No patient was eliminated. |
| Selective reporting (reporting bias) | Low risk | Comment: Study protocol was not available to identify any other unreported outcomes. Outcome mentioned on methods was reported in the results. |
| Other bias | Low risk | Comment: None were identified. |

**2.7. Meena *et al.*, 2020 (30)**

| **Bias** | **Authors' judgement** | **Support for judgement** |
| --- | --- | --- |
| Random sequence generation (selection bias) | Low risk | Quote: "... using a table of random numbers by using graph pad software." |
| Allocation concealment (selection bias) | Unclear risk | Comment: Allocation concealment was not specified. |
| Blinding of participants and personnel (performance bias) | Low risk | Quote: "Only one second year resident (who was posted in the wards and not in the NICU for the period of study) knew the codes of the neonates who received the drug Udcament and had no contact with either the parents or the medical team till the end of the study" |
| Blinding of outcome assessment (detection bias) | Low risk | Quote: "Only one second year resident (who was posted in the wards and not in the NICU for the period of study) knew the codes of the neonates who received the drug Udcament and had no contact with either the parents or the medical team till the end of the study" |
| Incomplete outcome data (attrition bias) | Unclear risk | Comment: There were 47 lost to follow-up patients with no details specified (whether they were lost before or after the randomization). |
| Selective reporting (reporting bias) | Low risk | Comment: Study protocol was not available to identify any other unreported outcomes. Outcome mentioned on methods was reported in the results. |
| Other bias | Low risk | Comment: None were identified. |

**2.8. Shahramian *et al*., 2019 (31)**

| **Bias** | **Authors' judgement** | **Support for judgement** |
| --- | --- | --- |
| Random sequence generation (selection bias) | Low risk | Quote: "... were selected using convenience method and were randomly (https://www.randomizer.org)" |
| Allocation concealment (selection bias) | Unclear risk | Comment: Allocation concealment was not specified. |
| Blinding of participants and personnel (performance bias) | Low risk | Quote: "One trained physician was aware of neonates who received UDCA and did not have any contact with either the parents or the medical team (including the nurses)" |
| Blinding of outcome assessment (detection bias) | Low risk | Quote: "One trained physician was aware of neonates who received UDCA and did not have any contact with either the parents or the medical team (including the nurses)" |
| Incomplete outcome data (attrition bias) | Low risk | Comment: No patient was eliminated. |
| Selective reporting (reporting bias) | Low risk | Comment: Outcome mentioned on study protocol and methods was reported in the results. |
| Other bias | Low risk | Comment: None were identified. |

**Supplementary Table 3. GRADE Assessment**

| **Certainty assessment** | | | | | | | **№ of patients** | | **Effect** | | **Certainty** | **Importance** |
| --- | --- | --- | --- | --- | --- | --- | --- | --- | --- | --- | --- | --- |
| **№ of studies** | **Study design** | **Risk of bias** | **Inconsistency** | **Indirectness** | **Imprecision** | **Other considerations** | **ursodeoxycholic acid** | **usual therapy** | **Relative (95% CI)** | **Absolute (95% CI)** |  |  |
| **Duration of phototherapy based on risk of bias** | | | | | | | | | | | | |
| 2 | randomised trials | not serious | not serious | not serious | not serious | none | 50 | 50 | - | MD 17.82 hours lower (20.17 lower to 15.47 lower) | ⨁⨁⨁⨁ High |  |
| **Duration of phototherapy** | | | | | | | | | | | | |
| 4 | randomised trials | not serious | serious^b^ | not serious | serious^c^ | none | 350 | 350 | - | MD 16.36 hours lower (26.21 lower to 6.51 lower) | ⨁⨁◯◯ Low |  |
| **Serum bilirubin (Asian Subgroup) (follow-up: 48 hours)** | | | | | | | | | | | | |
| 2 | randomised trials | not serious | not serious | not serious | serious^a^ | none | 140 | 140 | - | MD 0.43 mg/dL lower (0.64 lower to 0.22 lower) | ⨁⨁⨁◯ Moderate |  |
| **Serum bilirubin (follow-up: 48 hours)** | | | | | | | | | | | | |
| 3 | randomised trials | not serious | serious^b^ | not serious | serious^d^ | none | 190 | 190 | - | MD 0.54 mg/dL lower (0.91 lower to 0.18 lower) | ⨁⨁◯◯ Low |  |
| **Serum bilirubin (Asian subgroup) (follow-up: 24 hours)** | | | | | | | | | | | | |
| 4 | randomised trials | not serious | serious^b^ | not serious | serious^c^ | none | 350 | 350 | - | MD 1.87 mg/dL lower (3.40 lower to 0.35 lower) | ⨁⨁◯◯ Low |  |
| **Serum bilirubin (follow-up: 24 hours)** | | | | | | | | | | | | |
| 5 | randomised trials | not serious | serious^b^ | not serious | serious^e^ | none | 400 | 400 | - | MD 1.66 mg/dL lower (2.83 lower to 0.48 lower) | ⨁⨁◯◯ Low |  |
| **Serum bilirubin (follow-up: 36 hours)** | | | | | | | | | | | | |
| 3 | randomised trials | not serious | serious^b^ | not serious | not serious | none | 200 | 200 | - | MD 1.59 mg/dL lower (3.58 lower to 0.40 higher) | ⨁⨁⨁◯ Moderate |  |
| **Serum bilirubin (follow-up: 12 hours)** | | | | | | | | | | | | |
| 4 | randomised trials | not serious | serious^b^ | not serious | serious^c^ | none | 350 | 350 | - | MD 1.1 mg/dL lower (2.96 lower to 0.77 higher) | ⨁⨁◯◯ Low |  |

**CI:** confidence interval

#### Explanations

a. One out of two studies showed different decisions at either end of confidence intervals.

b. High heterogeneity.

c. One out of five studies showed different decisions at either end of confidence intervals.

d. One out of three studies showed different decisions at either end of confidence intervals.

e. One out of five studies showed different decisions at either end of confidence intervals.
